# Epigenetics, Oxidative Stress, and the Microbiome in Endometriosis: Toward an Integrated Mechanistic Framework for Precision Medicine

**DOI:** 10.3390/jpm16060299

**Published:** 2026-06-01

**Authors:** Nektaria Zagorianakou, Stylianos Makrydimas, Efthalia Moustakli, Emmanouil D. Oikonomou, Ioannis Mitrogiannis, Eleni Sintou, George Makrydimas

**Affiliations:** 1 Scientific Laboratory for Innovative Technologies in Internal Medicine, Department of Nursing, School of Health Sciences, University of Ioannina, 45500 Ioannina, Greece; zagorianakou@uoi.gr; 2 Medical School, Aristotle University of Thessaloniki, 54124 Thessaloniki, Greece; smakrydimas@gmail.com; 3 Department of Nursing, School of Health Sciences, University of Ioannina, 45500 Ioannina, Greece; ef.moustakli@uoi.gr; 4 Human Computer Interaction Laboratory, Department of Informatics and Telecommunications, University of Ioannina, 47150 Arta, Greece; e.oikonomou@uoi.gr; 5 Harris Birthright Research Centre for Fetal Medicine, King’s College London, London SE5 8BB, UK; ioannismitrogiannisgr@gmail.com; 6 Department of Anesthesia, Patras University Hospital, 26504 Patras, Greece; nelisintou@gmail.com; 7 Department of Obstetrics & Gynecology, University Hospital of Ioannina, 45500 Ioannina, Greece

**Keywords:** progesterone resistance, non-coding RNA regulation, DNA methylation patterns, immune dysregulation, fibrotic remodeling angiogenic mechanisms

## Abstract

Endometriosis (EM) is a chronic, estrogen-dependent inflammatory disorder affecting approximately 6–10% of women of reproductive age in the general population and remains a major cause of chronic pelvic pain and infertility. High recurrence rates and enduring symptoms despite current treatments underscore the need for a more thorough understanding of its intricate biology. There is growing evidence that the interaction among oxidative stress (OS), microbiome dysbiosis, and epigenetic dysregulation contributes to immunological activation, hormonal imbalance, and the persistence of ectopic lesions. Important disease mechanisms, such as progesterone resistance, inflammatory signaling, and aberrant cellular proliferation, are influenced by epigenetic changes, which include aberrant DNA methylation, histone modifications, and dysregulated non-coding RNAs. Simultaneously, high levels of reactive oxygen species (ROS) reinforce lesion survival and chronic inflammation by promoting angiogenesis, fibrosis, and tissue damage. Changes in the microbiome also affect immunological responses, oxidative balance, estrogen metabolism, and epigenetic control, indicating the existence of interrelated pathogenic loops. This narrative review presents an integrated mechanistic framework for endometriosis, summarizing the available data that connect these pathways. Furthermore, the growing implications of non-invasive biomarkers and precision medicine techniques highlight the potential for improved diagnosis, disease classification, and targeted treatment approaches.

## 1. Introduction

The presence of endometrium-like tissue outside the uterine cavity is the hallmark of EM, a chronic estrogen-dependent inflammatory disease that typically affects the ovaries, pelvic peritoneum, and rectovaginal septum. It is one of the main causes of infertility and chronic pelvic pain, affecting 6–10% of women of reproductive age globally [1,2]. Despite its high incidence, EM is still underdiagnosed, and diagnostic delays have been reported to range from approximately 4 to 11 years across different populations and study designs, which exacerbates long-term morbidity and disease progression [3,4,5].

The clinical manifestation of EM is highly diverse, ranging from severe pain syndromes and reproductive dysfunction to asymptomatic disease. Dysmenorrhea, non-cyclical chronic pelvic pain, dyspareunia, dyschezia, bowel and urinary dysfunction, exhaustion, and infertility are common symptoms that frequently manifest in complex and overlapping patterns [6]. Diagnosis, risk assessment, and therapy selection are severely hampered by this variability, which reflects underlying biological diversity. Beyond its physical manifestations, EM has a significant detrimental effect on mental health, social functioning, and overall quality of life. It also results in substantial socioeconomic consequences, including medical expenses, reduced workplace productivity, and absenteeism [3,6].

EM is also characterized by marked phenotypic heterogeneity. Distinct disease subtypes, including superficial peritoneal lesions, ovarian endometriomas, and deep infiltrating endometriosis (DIE), differ in their anatomical distribution, molecular features, and clinical behavior. In addition, clinical phenotypes such as pain-dominant and infertility-associated disease further reflect underlying biological variability and may influence disease progression and therapeutic response.

The majority of current endometriosis treatment approaches focus on symptom management rather than modifying the underlying disease. These include non-steroidal anti-inflammatory medications, hormonal treatments targeted at inhibiting estrogen signaling, surgical removal of lesions, and assisted reproductive technologies for the treatment of infertility. However, insufficient symptom relief, side effects, contraindications in women seeking pregnancy, and high recurrence rates—reported to reach up to 40–50% within five years after surgery—often limit these treatments, although recurrence varies depending on disease stage, lesion type, and surgical approach [7,8]. The absence of reliable biomarkers for early diagnosis, prognosis, and treatment response further underscores the need for a more comprehensive mechanistic understanding of EM pathophysiology.

Endometriosis is now widely recognized as a complex, multifactorial disease resulting from interactions among environmental exposures, immunological dysregulation, endocrine imbalance, genetic susceptibility, chronic inflammation, and angiogenesis [9,10]. The disease’s heritable and environmentally responsive characteristics may be explained by epigenetic mechanisms, such as DNA methylation, histone modifications, and non-coding RNAs, which modulate gene expression without changing the DNA sequence. The defining characteristics of EM, including progesterone resistance, immunological escape, inflammatory pathways, and estrogen signaling, are increasingly linked to these epigenetic changes [11].

Concurrently, oxidative stress (OS) has emerged as a major pathogenic factor in the development and progression of endometriosis. Excessive generation of ROS, together with weakened antioxidant defenses, promotes inflammation, cellular proliferation, fibrosis, and lesion persistence. Additionally, OS is a potent epigenetic modulator that reinforces pathogenic gene-expression programs in endometriotic tissue by altering DNA methylation, histone remodeling, and microRNA (miRNA) expression [12,13].

Recent studies identify the gut, reproductive tract, and peritoneal microbiota as key regulators of immunological homeostasis, estrogen metabolism, and inflammatory signaling in endometriosis. Increased intestinal permeability, compromised immune surveillance, altered estrogen recycling via the estrobolome, and persistent inflammatory activation are all possible consequences of dysbiosis. Notably, the microbiota, OS, and epigenetic regulation interact bidirectionally, indicating the presence of self-sustaining pathogenic loops that drive disease initiation and progression [14,15].

Collectively, these emerging lines of evidence suggest that EM is a systemic syndrome driven by interconnected molecular, metabolic, and microbial networks rather than a localized gynecologic disorder. However, current research often examines the microbiome OS, and epigenetics in isolation, thereby limiting the translation of mechanistic insights into clinical applications [16,17,18].

This narrative review synthesizes current evidence on epigenetic regulation, OS, and microbiome alterations in EM and proposes a unified mechanistic framework to explain disease heterogeneity and progression. To advance precision medicine in the treatment of EM, this review aims to facilitate the development of biomarker-driven diagnostics and tailored therapy approaches by emphasizing the dynamic connections among these pathways.

## 2. Literature Search Strategy

This narrative review was informed by a structured literature search focused on studies investigating epigenetic regulation, OS, immune dysregulation, and microbiome alterations in EM. The literature search was conducted using PubMed/MEDLINE, Scopus, and Web of Science databases. The final search was performed in July 2025, and the search timeframe included articles published between January 2000 and July 2025.

Search terms included combinations of the keywords “endometriosis,” “epigenetics,” “DNA methylation,” “histone modifications,” “microRNA,” “oxidative stress,” “reactive oxygen species,” “microbiome,” “dysbiosis,” “estrobolome,” “immune activation,” and “inflammation.” Boolean operators (AND/OR) were used to combine terms appropriately across databases.

Only articles published in English were considered. Eligible studies included original human studies, mechanistic experimental studies, translational investigations, systematic reviews, and relevant narrative reviews addressing molecular mechanisms, inflammatory pathways, oxidative imbalance, microbiome alterations, epigenetic regulation, biomarkers, or precision medicine approaches in EM. Studies unrelated to EM, non-peer-reviewed articles, conference abstracts without sufficient methodological detail, and studies lacking mechanistic or clinical relevance were excluded.

Priority was given to recent and methodologically robust studies, particularly those providing mechanistic insights, translational relevance, or integrative perspectives linking microbiome dysbiosis, OS, immune activation, and epigenetic dysregulation. Additional relevant articles were identified through manual screening of reference lists from selected publications.

Because this work represents a narrative rather than a systematic review, formal risk-of-bias assessment and quantitative meta-analysis were not performed. Instead, findings were qualitatively synthesized to develop an integrated mechanistic framework describing the interconnected biological pathways involved in EM.

## 3. Epigenetic Mechanisms in EM

The term “epigenetics” refers to heritable and reversible changes that regulate gene expression without altering the underlying DNA sequence. These processes primarily involve non-coding RNAs, especially miRNAs, post-translational histone modifications, and DNA methylation. Epigenetic dysregulation serves as a dynamic interface between environmental exposures, hormonal signaling, immunological activation, and aberrant cellular proliferation. Increasing evidence suggests that it plays a central role in the pathophysiology of EM. Epigenetic modifications are more malleable and responsive to internal and external stimuli than fixed genetic alterations [19,20]. Consequently, they are particularly relevant to the chronic and variable biological behavior of ectopic endometrial lesions. Additionally, such plasticity underpins the molecular basis of disease heterogeneity, progression, and therapeutic resistance.

### 3.1. DNA Methylation Abnormalities: Implantation Failure, Hormone Resistance, and Lesion Survival

Hypermethylation of the HOXA10 promoter, a master regulator of uterine receptivity, is one of the most frequently reported epigenetic changes. HOXA10 downregulation impairs endometrial differentiation, embryo implantation, and fertility potential [21]. Similarly, promoter hypermethylation of PGR-B, the progesterone receptor isoform required for stromal decidualization, contributes to progesterone resistance and impaired antiproliferative signaling within endometriotic tissue [22,23]. Aberrant methylation of ESR1 (ERα) further alters estrogen responsiveness, reinforcing local estrogen dominance and inflammatory signaling [17,24]. Collectively, these methylation abnormalities provide a mechanistic explanation for infertility, persistent lesion activity, and variable therapeutic responsiveness in EM.

Genome-wide methylation studies further demonstrate differential methylation patterns in genes regulating cell-cycle control (CDKN2A, CDKN1A), angiogenesis (VEGF), extracellular matrix remodeling (MMPs, TIMP3), and immune signaling pathways, including IL6 and CXCL12 [25,26]. These findings suggest that aberrant DNA methylation affects multiple interconnected biological systems involved in lesion establishment and progression rather than isolated hormone-signaling pathways alone.

However, these findings should be interpreted cautiously. Several foundational methylation studies in EM were conducted in relatively small and heterogeneous cohorts, with variability in lesion subtype, disease stage, and tissue sampling methodology. Such heterogeneity may limit reproducibility and generalizability, underscoring the need for larger, standardized, and well-characterized validation studies.

### 3.2. Histone Modifications: Shaping Chromatin Architecture and Inflammatory Gene Expression

Chromatin accessibility and transcriptional activity in endometriotic tissues are strongly influenced by histone alterations. Both eutopic and ectopic endometrium have been shown to exhibit changes in histone marks such as H3K27 trimethylation (H3K27me3), H3K9 acetylation (H3K9ac), and H4K16 acetylation (H4K16ac), which affect genes related to cell proliferation, inflammatory signaling, fibrosis, and steroid hormone responsiveness [27,28]. These modifications indicate a globally altered chromatin landscape that sustains persistent gene activation in endometriotic lesions.

These effects are further amplified by dysregulated expression of histone-modifying enzymes. Overexpression of histone acetyltransferases such as p300/CBP, enhancer of zeste homolog 2 (EZH2), and histone deacetylases (HDAC1/2) creates a permissive chromatin state that facilitates cellular invasion, increases inflammatory cytokine production, and promotes epithelial–mesenchymal transition [29]. HDAC overexpression has also been associated with fibrotic lesion formation, stromal cell contractility, and abnormal matrix remodeling. The therapeutic potential of targeting histone-modifying enzymes has therefore been highlighted in EM. In animal models, pharmacological inhibition of HDACs has demonstrated significant antiproliferative, anti-inflammatory, and anti-fibrotic effects [19,30].

### 3.3. miRNA Dysregulation: Post-Transcriptional Silencing and Inflammatory Amplification

MiRNAs are small non-coding RNAs that bind to target mRNAs and regulate gene expression post-transcriptionally by repressing translation or promoting mRNA degradation. Because they can regulate multiple genes simultaneously, miRNAs are powerful modulators of complex biological networks and have emerged as important contributors to the pathophysiology of EM. Several studies have demonstrated aberrant miRNA expression profiles in eutopic and ectopic endometrial tissue, as well as in circulating biofluids of affected individuals [31].

For example, overexpression of miR-135a and miR-135b suppresses HOXA10 expression, thereby impairing embryo implantation and endometrial receptivity [32]. Persistent overexpression of miR-21 in endometriotic lesions promotes cellular proliferation and inhibits apoptosis by suppressing tumor suppressor pathways, including PTEN signaling [33]. miRNA dysregulation is also linked to immune activation and redox imbalance; for example, miR-451 has been shown to regulate OS pathways and inflammatory responses in other biological contexts [34]. Members of the miR-200 family regulate fibrotic remodeling and epithelial–mesenchymal transition, processes that are essential for lesion invasiveness and chronicity [35].

Notably, specific miRNA expression signatures have been proposed as potential non-invasive biomarkers for EM, enabling identification of affected individuals using endometrial, serum, or plasma samples. Beyond their diagnostic potential, miRNAs also represent promising therapeutic targets, as modulation of their expression may simultaneously correct multiple pathological pathways [36,37].

However, findings on circulating miRNA signatures remain inconsistent across studies. Variability in sample collection, menstrual cycle phase, and pre-analytical factors such as haemolysis can significantly influence miRNA profiles. A systematic evaluation by Nisenblat et al., (2019) reported limited diagnostic accuracy of circulating miRNAs, highlighting the need for standardisation and validation in larger, well-controlled cohorts [38].

### 3.4. Epigenetic Regulation of Immune–Endocrine Crosstalk

EM is characterized by persistent low-grade inflammation, reduced natural killer cell cytotoxicity, and macrophage activation, suggesting a strong epigenetic basis for immune dysfunction. Aberrant DNA methylation and histone modifications in genes regulating cytokines (e.g., TNF, IL1B), chemokines, and immune receptors drive persistent inflammatory signaling and immune evasion within the peritoneal environment [39,40]. These epigenetic alterations create a tolerogenic immune milieu that permits the survival and growth of ectopic endometrial cells.

Steroid hormones also interact bidirectionally with epigenetic machinery. While epigenetic changes enhance estrogen dependency and progesterone resistance, estrogen signaling regulates the activity of DNA methyltransferases and histone-modifying enzymes, thereby reinforcing epigenetic reprogramming. This reciprocal interaction contributes to the autonomous activity of endometriotic lesions, which is frequently uncoupled from systemic hormone levels [41,42].

### 3.5. Environmental Influences on the Epigenome

Environmental exposures play an important role in modulating epigenetic regulation in EM. Endocrine-disrupting chemicals (EDCs), including dioxins, bisphenol A (BPA), and polychlorinated compounds, have been shown to induce persistent epigenetic alterations in reproductive tissues. Exposure to environmental toxins such as 2,3,7,8-Tetrachlorodibenzo-p-dioxin (TCDD) has been linked to alterations in DNA methylation and histone modifications in genes involved in hormonal signaling, immune regulation, and inflammatory pathways [43,44]. These findings support the hypothesis that environmental exposures, possibly occurring in early life or even during fetal development, may predispose susceptible individuals to EM through epigenetic reprogramming.

### 3.6. Epigenetic Plasticity as a Therapeutic Opportunity

**Figure 1:**
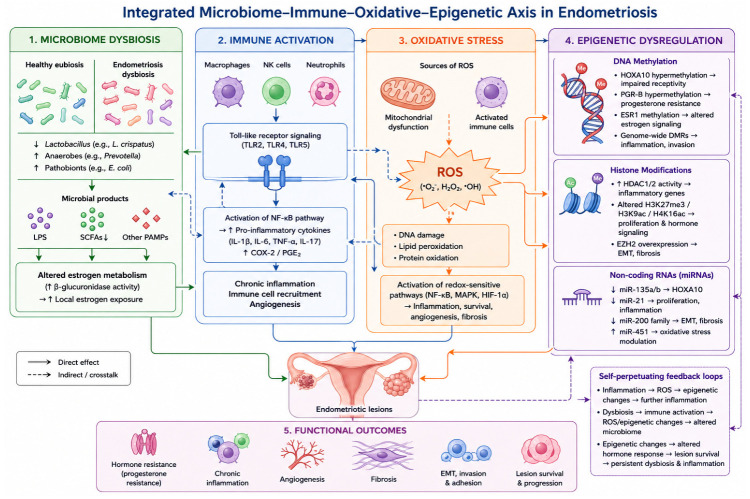
Integrated microbiome–immune–oxidative–epigenetic axis in EM. Schematic representation of how dysbiosis, immune activation, OS, and epigenetic changes interact to promote lesion implantation, inflammation, and disease progression. Abbreviations: EM, endometriosis; miRNA, microRNA; HOXA10, Homeobox A10; PGR-B, progesterone receptor B isoform; ESR1 (ERα), estrogen receptor alpha; DMRs, differentially methylated regions; HDAC1/2, histone deacetylases 1 and 2; H3K27me3, trimethylation of histone H3 lysine 27; H3K9ac, acetylation of histone H3 lysine 9; H4K16ac, acetylation of histone H4 lysine 16; EZH2, enhancer of zeste homolog 2; EMT, epithelial–mesenchymal transition.

Because epigenetic changes are reversible, they represent attractive therapeutic targets in EM. Emerging strategies include miRNA-based therapies, DNA methyltransferase inhibitors, and histone deacetylase inhibitors designed to modulate epigenetic regulators. Most evidence supporting epigenetic-targeted therapies currently derives from preclinical and experimental models, whereas robust clinical validation in human cohorts remains limited [19,45]. Epigenetic therapies, therefore, show promise as disease-modifying treatment strategies. Table 1 summarizes the major epigenetic processes implicated in EM and their functional implications. Figure 1 provides a systems-level overview of the integrated microbiome–immune–oxidative–epigenetic axis contributing to lesion establishment, inflammation, and progression in EM.

## 4. OS and Its Role in Disease Progression

EM is initiated, maintained, and progresses largely due to OS. OS occurs when excessive production of ROS overwhelms intracellular antioxidant defense systems. This imbalance leads to oxidative DNA damage, lipid peroxidation, protein carbonylation, mitochondrial dysfunction, and enhanced inflammatory signaling [13,60]. Elevated oxidative byproducts, disturbed redox homeostasis, and decreased detoxification capacity are routinely observed in the peritoneal microenvironment of women with EM. These changes establish OS as a unifying pathogenic factor rather than merely a downstream consequence of inflammation by supporting ectopic lesion survival, invasion, and symptom persistence within a prolonged, self-sustaining inflammatory loop [13,61].

### 4.1. Major Sources of ROS in EM

In EM, increased production of ROS within the peritoneal and lesional milieu arises from multiple endogenous and environmental factors. Retrograde menstruation, which introduces iron, free heme, and erythrocytes into the peritoneal cavity, represents one of the earliest and most significant contributors. Highly reactive hydroxyl radicals are generated when activated macrophages degrade hemoglobin, releasing ferrous iron (Fe^2+^), a powerful catalyst of the Fenton and Haber–Weiss reactions. Iron accumulation within the peritoneal environment is increasingly recognized as a central driver of oxidative damage and lesion persistence in EM [62]. These radicals promote genomic instability and increase the survival and implantation potential of ectopic endometrial cells by inducing lipid peroxidation, reflected by elevated malondialdehyde and 4-hydroxynonenal levels, as well as oxidative DNA damage and strand breaks [63,64,65]. Iron-induced oxidative damage also promotes fibrotic remodeling and epithelial–mesenchymal transition, two processes closely associated with lesion invasiveness and disease progression.

Concurrently, OS in EM is significantly amplified by immunological dysregulation. Activated macrophages abundant in the peritoneal cavity of affected women secrete numerous pro-inflammatory cytokines, including tumor necrosis factor-α, interleukin-1β, interleukin-6, interleukin-8, and prostaglandins. These mediators promote mitochondrial ROS leakage in immune and stromal cells, and enhance OS primarily through activation of NADPH oxidase pathways [66,67]. Inflammatory cytokines also stimulate neutrophil recruitment and respiratory burst activity while suppressing endogenous antioxidant defenses such as glutathione peroxidase, catalase, and superoxide dismutase. Immune-mediated oxidative amplification, therefore, sustains a chronic inflammatory environment that favors the persistence of ectopic lesions and resistance to immune clearance.

Another intrinsic source of OS arises from endometriotic stromal cells themselves. These cells exhibit excessive production of superoxide anions and hydrogen peroxide as a result of mitochondrial dysfunction, characterized by altered electron transport chain activity and reduced mitochondrial membrane integrity [68,69]. The accumulation of mitochondrial DNA mutations, resembling a Warburg-like metabolic phenotype described in proliferative diseases, may contribute to metabolic reprogramming toward aerobic glycolysis in endometriotic cells. Although antioxidant mechanisms such as the NRF2 signaling axis often display compensatory activation, they remain insufficient to counterbalance the persistent oxidative burden. Consequently, mitochondria contribute to cellular dysfunction and lesion survival by acting as both sources and targets of ROS [70,71].

The oxidative landscape of EM is further influenced by metabolic and environmental factors. Exposure to endocrine-disrupting chemicals such as dioxins, phthalates, and bisphenol A has been shown to increase ROS production and alter inflammatory and immune responses in the peritoneal environment [72,73]. In addition, systemic metabolic disorders, including obesity, dyslipidemia, and hyperinsulinemia, elevate baseline OS and may exacerbate lesion activity and symptom severity. Together, these convergent factors create a highly pro-oxidant environment that accelerates disease development, tissue remodeling, and inflammation [74,75].

### 4.2. OS–Epigenetic Interactions: A Bidirectional Regulatory Network

In EM, OS and epigenetic dysregulation form an interconnected molecular network that reinforces disease progression. Beyond direct cellular damage, excessive ROS modify the epigenome, thereby amplifying oxidative and inflammatory pathways [76,77,78].

ROS-induced oxidation of guanine to 8-oxo-deoxyguanosine disrupts DNA methyltransferase binding and activity, resulting in abnormal DNA methylation patterns. OS has been linked to increased DNMT1 activity in EM, resulting in hypermethylation and silencing of key genes such as HOXA10 and PGR and contributing to progesterone resistance and implantation failure [76,77]. Conversely, hypomethylation of promoters regulating pro-inflammatory cytokines sustains persistent transcriptional activation and immune amplification.

OS also modulates histone modifications by altering the activity of chromatin-modifying enzymes. The redox sensitivity of histone deacetylases allows elevated OS to increase their expression and activity, thereby promoting chromatin condensation and repression of anti-inflammatory and anti-proliferative genes [78,79]. At the same time, transcription of inflammatory mediators is facilitated by ROS-mediated activation of histone acetyltransferases such as p300/CBP. In addition, lipid peroxidation products such as 4-hydroxynonenal can directly bind histone proteins, altering chromatin architecture and nucleosome stability [80].

By impairing Drosha and Dicer complexes, OS also disrupts miRNA processing and alters miRNA expression profiles. Downregulation of the miR-200 family promotes epithelial–mesenchymal transition and invasiveness, upregulation of miR-21 supports fibrosis and cellular survival, and altered expression of miR-451 regulates inflammatory and antioxidant pathways [35,56]. These changes reinforce the invasive, inflammatory, and proliferative characteristics of endometriotic lesions.

### 4.3. Clinical Relevance of OS in EM

OS directly influences symptom severity and disease progression in EM. Elevated ROS levels contribute to pain generation by increasing prostaglandin synthesis, promoting neuroinflammation, and sensitizing peripheral nociceptors. The association between OS markers and pain severity is supported by increased nerve fiber density observed in endometriotic lesions exhibiting high oxidative activity [81,82].

Infertility associated with EM is also strongly influenced by OS. Excess ROS destabilizes luteal phase function, impairs endometrial receptivity, damages tubal function, and compromises oocyte quality. Elevated oxidative burden within the peritoneal environment further reduces reproductive potential by negatively affecting sperm motility, fertilization capacity, and early embryo development [83,84].

At the tissue level, ROS promote vascular endothelial growth factor-mediated angiogenesis, activate matrix metalloproteinases that facilitate invasion, and stimulate transforming growth factor-β/SMAD signaling pathways that drive fibrosis [85]. These mechanisms support lesion formation, promote the development of deep infiltrating EM, and contribute to progressive organ damage. These clinically relevant features of EM reflect stable epigenetic programs reinforced by OS, as summarized in Table 2.

### 4.4. Antioxidant-Based Therapeutic Approaches

Antioxidant-based therapies have attracted growing interest as adjunctive treatments, as OS plays a central role in the pathogenesis of EM. Compounds such as vitamins C and E, melatonin, N-acetylcysteine, omega-3 fatty acids, and resveratrol have shown potential in preclinical and early clinical studies to reduce ROS levels and modulate inflammatory signaling [87,88]. In particular, N-acetylcysteine increases intracellular glutathione levels, while melatonin exhibits antioxidant and analgesic properties [88,89].

However, despite encouraging preliminary findings, antioxidant-based interventions in EM remain investigational because existing studies are limited by heterogeneous methodologies, inconsistent outcome measures, and relatively small patient cohorts. Future large-scale and standardized clinical trials are required to clarify their therapeutic value and potential integration into precision medicine approaches discussed in Section 7.3.

## 5. Microbiome Alterations in EM

The microbiome is increasingly recognized as a key modulator of the immunological, endocrine, metabolic, and epigenetic networks implicated in the pathogenesis of EM [14,90]. Dysbiosis has been reported in the gastrointestinal system, peritoneal cavity, and reproductive tract, indicating a systemic microbial signature rather than isolated local alterations. These microbial changes establish a multidirectional mechanistic axis that facilitates lesion implantation, survival, and progression through interactions with immune activation, OS, estrogen metabolism, and epigenetic reprogramming [91].

### 5.1. Reproductive-Tract Dysbiosis

Several studies show that, compared with healthy controls, women with EM exhibit altered microbial profiles in the vagina, cervix, and endometrium. Lactobacillus species—especially *Lactobacillus crispatus* and *Lactobacillus jensenii*—which normally maintain an acidic environment, reinforce epithelial barrier integrity, and inhibit pathogenic colonization, have been reported to be reduced in several studies [92,93]. This reported reduction in Lactobacillus dominance has been associated with increased abundance of facultative anaerobic and pro-inflammatory taxa such as *Gardnerella*, *Atopobium*, *Streptococcus*, and members of the *Escherichia/Shigella* group, which are commonly associated with inflammatory reproductive-tract environments [94,95]. Women with EM also exhibit alterations in the Firmicutes-to-Bacteroidetes ratio, resembling patterns of gut dysbiosis associated with systemic inflammation [96].

Disruption of the protective Lactobacillus-dominated microbiota increases epithelial permeability, facilitating exposure to pathogen-associated molecular patterns (PAMPs). Activation of Toll-like receptor-mediated signaling pathways, particularly via TLR4, stimulates the secretion of pro-inflammatory cytokines by macrophages, including tumor necrosis factor-α, interleukin-1β, and interleukin-6 [97,98]. Lipopolysaccharides produced by Gram-negative bacteria further ROS production, creating a pro-oxidant inflammatory pelvic environment that facilitates lesion formation and persistence.

However, it should be noted that microbiome findings in endometriosis are heterogeneous across studies. Variability in sampling sites (vaginal, endometrial, peritoneal, or fecal), sequencing methodologies, and population characteristics contributes to differences in reported microbial profiles. In addition, many currently available data derive from relatively small observational cohorts and remain insufficiently externally validated across phenotypically stratified patient populations. As a result, a consistent disease-specific microbiome signature has not yet been fully established, and current findings should be interpreted with caution.

### 5.2. Gut Microbiome, Estrogen Metabolism, and the Estrobolome

The gut microbiome regulates systemic estrogen levels through the estrobolome, a collection of bacterial genes encoding β-glucuronidase enzymes [99]. These enzymes can deconjugate estrogens following hepatic conjugation and biliary excretion, allowing enterohepatic reabsorption and increasing systemic estrogen bioavailability. During dysbiosis, increased abundance of Enterobacteriaceae elevates β-glucuronidase activity, thereby promoting estrogen-dependent lesion formation [99,100]. Concurrently, reduced levels of beneficial genera such as Lactobacillus and Bifidobacterium impair anti-inflammatory regulation and epithelial integrity, exacerbating immune and endocrine dysregulation [101]. These findings highlight the gut microbiota as a key endocrine regulator in the pathophysiology of EM.

### 5.3. Microbiome–Immune–Epigenetic Crosstalk

Microbiome-driven mechanisms in EM involve immune activation, epigenetic alterations, and endocrine modulation. Bacterial components, including lipopolysaccharides and microbial metabolites, enter the systemic circulation as a result of increased intestinal permeability associated with gut dysbiosis. This endotoxemia activates innate immune signaling pathways, particularly via TLR4, leading to macrophage activation, increased secretion of tumor necrosis factor-α, interleukin-6, and interleukin-8, and stimulation of NADPH oxidase-mediated OS [91,102,103]. Recruitment of neutrophils and dendritic cells further amplifies inflammatory signaling, contributing to increased lesion invasiveness, neuroangiogenesis, and persistent inflammation.

Microbial metabolites also influence epigenetic regulation. Commensal gut bacteria produce short-chain fatty acids, including butyrate, acetate, and propionate, which function as natural histone deacetylase inhibitors and play important roles in immune tolerance and anti-inflammatory gene expression [104,105]. Reduced abundance of short-chain fatty acid-producing taxa such as Roseburia and Faecalibacterium has been associated with diminished anti-inflammatory signaling and abnormal DNA methylation patterns in EM [106]. Through these mechanisms, dysbiosis can directly influence epigenetic reprogramming in immune and endometrial cells.

OS pathways also interact with dysbiotic microbial populations. ROS may be produced directly by certain microbial taxa or indirectly through immune activation. OS, therefore, creates a self-reinforcing loop linking dysbiosis, inflammation, OS, and epigenetic dysfunction by inducing DNA damage, altering DNA methylation patterns, and modulating the activity of DNA methyltransferases and histone deacetylases [107,108,109].

### 5.4. The Infection Hypothesis

A complementary mechanistic hypothesis proposes that specific microbial infections may promote or exacerbate the oxidative and inflammatory conditions required for lesion development. Innate immune receptors such as TLR4 and TLR5 are activated by microbial components, including lipopolysaccharide (LPS), flagellin, and peptidoglycan. This activation promotes aromatase activity, cyclooxygenase-2 overexpression, prostaglandin E2 synthesis, and downstream NF-κB signaling, ultimately increasing local estrogen production and supporting lesion survival and proliferation [110,111].

Consistent with this hypothesis, bacterial taxa such as *Escherichia coli*, *Streptococcus*, and *Peptostreptococcus* have been reported to be more abundant in the peritoneal fluid of women with EM and may act as initiating or amplifying microbial triggers [18,112].

### 5.5. Integrative Model of Microbiome-Driven Pathophysiology

Current evidence supports a multidirectional mechanistic model in which immune activation drives OS, leading to tissue damage and increased implantation potential. In parallel, increased intestinal permeability facilitates translocation of bacterial LPS into the circulation, while dysbiosis promotes the expansion of estrogen-modulating and pro-inflammatory microbial taxa. Estrobolome-mediated estrogen recycling maintains a hormonal environment favorable for lesion survival, while microbial metabolites simultaneously influence epigenetic programs that promote angiogenesis, fibrosis, and epithelial–mesenchymal transition [102,113,114].

This integrative paradigm aligns with the gene–microbiome–epigenetic axis proposed in recent studies of the gut–EM relationship [60]. The principal microbiome-related alterations and their effects on OS, immune activation, and epigenetic regulation in EM are summarized in Table 3.

## 6. Integrated Mechanistic Model

EM emerges from the dynamic interaction of three interdependent biological systems: epigenetic dysregulation, OS, and microbiome alterations. Rather than acting independently, these processes form interconnected, self-reinforcing feedback loops that sustain chronic inflammation, immune dysfunction, endocrine imbalance, and ectopic lesion persistence [123].

From a temporal perspective, these interactions can be broadly divided into initiation and maintenance phases. Early events, including retrograde menstruation, immune activation, and microbiome perturbations, contribute to a pro-inflammatory and pro-oxidative environment that facilitates lesion establishment, whereas sustained epigenetic and inflammatory signaling pathways support disease persistence and progression.

These processes converge to form a microbiome–immune–oxidative–epigenetic axis that sustains EM as a chronic, progressive disorder. Dysbiosis promotes immune activation and OS, which reshape the epigenome, while epigenetic alterations stabilize pro-inflammatory, estrogen-dependent gene expression programs that sustain lesion persistence [102,124]. These interactions are not linear but directional and self-reinforcing. Microbiome alterations can initiate immune activation and OS, OS can induce epigenetic reprogramming, and epigenetic changes can stabilize inflammatory and endocrine dysregulation, thereby reinforcing the initial triggers.

This reciprocal reinforcement provides a mechanistic explanation for key clinical features of EM, including symptom chronicity, disease recurrence following treatment, interindividual heterogeneity, and variable therapeutic response [125]. These mechanisms may differ across disease subtypes (e.g., peritoneal lesions, ovarian endometriomas, and deep infiltrating endometriosis) and clinical phenotypes, contributing to the biological and clinical heterogeneity of EM.

This integrative framework also generates testable predictions. For example, targeted modulation of the microbiome may alter epigenetic and inflammatory profiles in endometriotic tissue [126]. Similarly, antioxidant or redox-targeted interventions may partially reverse epigenetic dysregulation and improve hormonal responsiveness [127]. In addition, multi-omics biomarker panels integrating microbiome, epigenetic, and inflammatory signatures may improve the prediction of disease progression and recurrence compared with single-marker approaches [128].

This integrated mechanistic model aligns with emerging interdisciplinary evidence linking gut microbiota, OS, and epigenetic regulation in EM [129]. Validation of this integrative framework will require multi-omics approaches across independent and well-characterized patient cohorts. The systems-level interactions among these pathways are summarized in Table 4.

## 7. Biomarkers and Precision Medicine Applications

The integration of molecular biomarkers into clinical practice represents a promising approach toward improving diagnosis, disease stratification, and personalized therapeutic strategies, although most applications remain investigational in EM. Given the marked heterogeneity in clinical presentation, disease progression, and treatment response, single-marker approaches are unlikely to capture the full biological complexity of the disorder [132]. Instead, multi-omics strategies integrating genomics, epigenomics, transcriptomics, proteomics, metabolomics, and microbiome profiling provide a foundation for precision medicine approaches that address interindividual variability and identify biologically defined disease subtypes [133].

### 7.1. Genomic and Epigenomic Biomarkers

Epigenetic biomarkers in endometriosis can be broadly categorized into tissue-based markers and circulating biomarkers, with differing levels of clinical validation.

Among epigenomic biomarkers, hypermethylation of the HOXA10 promoter is one of the most consistently replicated findings in EM. This tissue-based alteration is strongly associated with impaired endometrial receptivity and infertility, reflecting disrupted decidualization and implantation failure [21,134]. Detection of HOXA10 methylation in endometrial tissue, and potentially through minimally invasive liquid biopsy approaches, may help identify patients at high risk of reproductive failure and potentially inform fertility counseling and treatment planning, although further validation is required [19].

Aberrant methylation of ESR1, encoding estrogen receptor α, represents another promising tissue-based biomarker. Altered ESR1 methylation modifies estrogen receptor expression and downstream signaling, contributing to local hyperestrogenism and persistence of ectopic lesions [135]. Assessment of ESR1 methylation status may therefore serve as a potential indicator of estrogen dependence, although its clinical utility remains under investigation.

Progesterone resistance, a hallmark of EM that limits the efficacy of progestin-based treatments, has also been linked to methylation of the PGR-B promoter. Reduced expression of this progesterone receptor isoform impairs anti-proliferative and anti-inflammatory signaling within endometrial tissue [23,42]. Epigenetic profiling of PGR methylation patterns may contribute to patient stratification in future applications according to predicted responsiveness to hormonal therapies and support more personalized treatment selection.

Beyond single-gene targets, dysregulated miRNA expression has emerged as a promising source of circulating, minimally invasive biomarkers. MiRNAs involved in inflammation, epithelial–mesenchymal transition, angiogenesis, and cell survival, including miR-21, miR-135a, miR-451, and members of the miR-200 family, have been reported to be altered in eutopic and ectopic endometrium as well as in circulating biofluids [136,137,138,139]. Composite circulating miRNA panels have shown variable but promising diagnostic performance. For example, several studies have identified miRNA signatures capable of distinguishing women with and without EM [140,141,142]. These profiles hold particular promise as liquid biopsy tools for non-invasive diagnosis and disease monitoring.

At a broader level, epigenome-wide association studies have identified differentially methylated regions in genes regulating immune responses, steroid hormone signaling, extracellular matrix remodeling, and cell-cycle control [41]. Such multi-marker epigenomic signatures may ultimately outperform single-gene assays by enabling more refined molecular classification of EM subtypes and supporting biologically informed risk stratification [143].

Importantly, a distinction must be made between mechanistically informative biomarkers and those with demonstrated clinical utility. While many candidate markers provide insight into disease pathophysiology, only a limited number have undergone sufficient validation for clinical application. Most epigenetic biomarkers remain investigational, with limited reproducibility across studies and are not yet implemented in routine clinical practice. Furthermore, many proposed biomarkers are tissue-based and therefore have limited applicability for non-invasive clinical diagnosis. Similar challenges apply to inflammatory and proteomic biomarkers derived from plasma and peritoneal fluid, where several candidate markers demonstrate mechanistic relevance but insufficient clinical validation and reproducibility for routine implementation.

### 7.2. Microbiome-Derived Biomarkers

Microbiome-derived biomarkers include both taxonomic signatures and metabolite-based markers, which are primarily investigational and vary in clinical applicability.

Microbiome profiling represents an emerging and complementary source of biomarkers in EM. In the reproductive tract, reduced dominance of Lactobacillus species and enrichment of genera such as *Gardnerella*, *Atopobium*, *Streptococcus*, and *Escherichia*/*Shigella* have been reported in women with EM and related gynecologic disorders [96]. These microbial patterns reflect a pro-inflammatory pelvic environment and may serve as candidate non-invasive biomarkers, particularly in relation to inflammatory burden and symptom severity.

At both the reproductive and intestinal levels, overrepresentation of pathogenic or pro-inflammatory taxa, including *Proteobacteria* and members of the *Enterobacteriaceae* family, has been associated with increased cytokine production, OS, and epithelial barrier dysfunction [91,96,102]. Incorporating such microbial signatures into clinical risk models may have the potential to improve the prediction of disease activity or recurrence, although validation remains limited.

Gut microbiome-derived biomarkers are particularly relevant in the context of estrogen metabolism. The altered abundance of β-glucuronidase-producing bacteria influences enterohepatic estrogen recycling and circulating estrogen levels [99]. Estrogen-related microbial markers may therefore help identify patients with strongly estrogen-dependent disease and may potentially guide the selection and intensity of hormonal or anti-estrogenic therapies, although their clinical application remains under investigation [99].

In addition to taxonomic profiles, microbial metabolite patterns provide functional biomarker information. Reduced levels of short-chain fatty acids, particularly butyrate and propionate, reflect depletion of beneficial genera such as *Faecalibacterium* and *Roseburia* and are associated with heightened inflammation and altered epigenetic regulation [141,142]. These metabolomic signatures may serve as indicators of disease severity, recurrence risk, or treatment resistance, although current evidence remains heterogeneous and requires further validation in larger patient cohorts.

Interpretation of molecular and microbiome-derived biomarkers in endometriosis is subject to several important limitations. Clinical and biological confounders, including menstrual cycle phase, hormonal therapy, disease stage, and prior surgical interventions, can significantly influence epigenetic, inflammatory, and microbial profiles. In addition, technical constraints must be considered. For example, 16S rRNA sequencing has limited resolution at the species and strain level, bulk tissue methylation analyses may obscure cell-type-specific epigenetic changes, and circulating miRNA assays show variability across platforms and limited reproducibility. Furthermore, variability in sampling methods, sequencing approaches, and bioinformatic pipelines currently limits standardization and comparability across microbiome studies. These factors currently limit the translation of candidate biomarkers into clinical practice and highlight the need for standardised protocols and large, well-characterised cohorts.

Despite promising mechanistic and translational findings, most epigenetic, microbiome-derived, and metabolomic biomarkers in EM remain investigational and are not currently ready for routine clinical implementation. Their reproducibility and diagnostic performance are limited by methodological heterogeneity, small cohort sizes, and variability related to disease phenotype, menstrual cycle phase, hormonal treatment exposure, and prior surgical history. Future validation studies using large, well-characterized, and phenotypically stratified patient cohorts will be essential before these biomarkers can be reliably incorporated into precision medicine algorithms and clinical decision-making.

### 7.3. Precision Medicine Opportunities

Although these approaches are promising, most precision medicine strategies in endometriosis remain at an early stage of development and require further clinical validation before routine implementation.

The integration of molecular biomarkers into clinical decision-making opens multiple avenues for precision medicine in EM. Biomarker-guided anti-inflammatory and antioxidant strategies represent one such opportunity. Nutritional interventions, including Mediterranean-style or anti-inflammatory diets, together with supplementation with omega-3 fatty acids, vitamins C and E, melatonin, and N-acetylcysteine, have been shown to reduce OS, alleviate pain, and modulate inflammatory markers in subsets of patients [144,145]. Identifying individuals with pronounced oxidative or inflammatory signatures may potentially enhance the effectiveness of these interventions.

Lifestyle and exercise interventions also represent modifiable components of personalized care. Regular physical activity influences systemic inflammation, redox balance, and gut microbial composition. Integrating clinical characteristics with inflammatory, OS, and microbiome biomarkers may support the future tailoring of lifestyle and exercise interventions to individual biological profiles, thereby maximizing benefits while minimizing symptom exacerbation [146].

Epigenetic-modifying therapies constitute another emerging precision strategy. Although several precision medicine strategies show promise in preclinical or early translational studies, few have undergone prospective validation in large human cohorts [147]. In the future, epigenetic signatures, such as HOXA10, ESR1, and PGR methylation status, may help identify patients most likely to benefit from such targeted approaches.

Microbiome-based interventions, including probiotics, prebiotics, synbiotics, and potentially fecal microbiota transplantation, aim to restore microbial balance, reinforce epithelial integrity, and reduce inflammation [148]. Personalized modulation of the microbiome according to individual dysbiosis patterns and estrobolome activity may represent a potential component of precision medicine approaches in EM [10].

Finally, computational precision-medicine platforms offer a unifying framework for integrating diverse data streams. Machine-learning models integrating genomic, epigenomic, microbiome, metabolomic, clinical, and lifestyle data may support the prediction of disease subtype, recurrence risk, and treatment response [149]. Such integrative tools may contribute to future clinical decision-support systems, enabling a transition from symptom-based management toward biologically informed, individualized care in EM. Bridging the gap between mechanistic insights and clinical implementation remains a major challenge in the field.

## 8. Limitations

This narrative review has several limitations. First, it does not follow a systematic review methodology, and selection bias may therefore occur despite a thorough and structured literature search. Second, much of the evidence linking OS, epigenetic alterations, and microbiome dysbiosis in EM derives from cross-sectional studies, small cohorts, and heterogeneous methodologies, particularly in microbiome and epigenomic analyses. Direct comparability between studies is limited by variations in sample types, analytical platforms, and disease classification, as well as differences in microbiome sampling procedures, sequencing technologies, and bioinformatic pipelines, which currently lack standardization. Third, although mechanistic insights from in vitro and animal models are valuable, their translation to human disease remains limited. Finally, longitudinal, interventional, and multi-omics validation studies across independent and well-characterized patient cohorts are required to establish causal relationships and clinical utility; thus, the integrated mechanistic framework presented here should be considered hypothesis-generating and reflective of current understanding. Nevertheless, the relevance of the proposed systems-level model is supported by the convergence of findings across multiple research fields.

## 9. Conclusions

Microbiome dysbiosis, OS imbalance, and epigenetic reprogramming contribute to EM, which is increasingly recognized as a complex, systems-level disorder. These intricately linked, self-reinforcing pathways shape lesion biology, immune–endocrine interactions, nociceptive signaling, and disease chronicity. Microbial disruptions influence estrogen metabolism, immune activation, redox homeostasis, and chromatin dynamics. OS intensifies inflammatory, angiogenic, and fibrotic pathways while further reshaping the epigenome. In turn, epigenetic alterations stabilize aberrant transcriptional programs that support progesterone resistance, inflammation, and tissue remodeling. Collectively, these mechanisms constitute a multifaceted pathogenic framework that helps explain the marked heterogeneity in clinical presentation, disease progression, and therapeutic response in EM.

Clarifying these interconnected biological networks is essential for advancing molecularly informed diagnostics, developing reliable non-invasive biomarkers, and implementing precision medicine tailored to individual pathophysiological profiles. Early diagnosis, improved risk assessment, and more durable disease modification may be achieved by shifting from symptom-based treatment toward systems-level, mechanism-driven strategies. The integration of multi-omics technologies and computational modeling into clinical practice has the potential to transform EM care into a personalized, predictive, and preventive paradigm.

## Figures and Tables

**Table 1:** Key DNA methylation changes, histone modifications, and dysregulated miRNAs reported in EM and their functional effects on endometrial receptivity, inflammation, fibrosis, and lesion development.

Epigenetic Mechanism	Key Molecular Findings	Functional Consequences
DNA Methylation [46,47]	HOXA10 promoter hypermethylation	Impaired endometrial receptivity, reduced implantation, infertility
[25,48]	PGR-B promoter hypermethylation	Progesterone resistance, impaired decidualization
[25,26]	ESR1 (ERα) promoter methylation	Altered estrogen responsiveness, enhanced local estradiol signaling
[26,49]	Differential methylation of immune–inflammatory genes (IL6, CXCL12)	Chronic inflammation, macrophage activation, immune evasion
[26,50]	Genome-wide DMRs in angiogenesis and ECM remodeling genes (VEGF, MMPs, TIMPs)	Lesion invasion, ECM dysregulation, fibrogenesis
Histone Modifications [19,51]	Increased HDAC1/2 activity	Enhanced inflammatory gene transcription, stromal cell invasiveness
[50,52]	Altered H3K27me3, H3K9ac, H4K16ac	Dysregulated proliferation, aberrant steroid signaling, angiogenesis
[53]	EZH2 overexpression	EMT activation, fibrosis, lesion survival
Non-Coding RNAs (miRNAs) [54]	↓ miR-135a/b → HOXA10 suppression	Impaired implantation, defective endometrial differentiation
[36]	↑ miR-21 overexpression	Increased proliferation, apoptosis inhibition, inflammation
[55]	↓ miR-200 family	EMT activation, invasiveness, fibrosis
[56]	↑ miR-451	Modulation of OS pathways, inflammatory amplification
[57,58]	Circulating/exosomal miRNA biomarker panels	Minimally invasive diagnostic potential
Environmental Epigenetic Influences [59]	TCDD/BPA-induced epigenetic alterations	Environmental priming, increased susceptibility

**Table 2:** This table summarizes major sources of OS in EM and their effects on DNA methylation, histone modifications, and non-coding RNA regulation, highlighting downstream consequences for inflammation, hormone resistance, fibrosis, and lesion persistence.

OS Source	Epigenetic Mechanism	Key Molecular Targets	Biological Outcome
Iron overload/heme degradation [47,86]	DNA hypermethylation	DNMT1, HOXA10, PGR	Progesterone resistance, implantation failure
ROS accumulation [13]	DNA oxidation (8-oxo-dG)	CpG-rich promoters	Aberrant methylation patterns
Chronic inflammation [51,52]	Histone deacetylation	HDAC1/2, NF-κB–regulated loci	Sustained inflammatory transcription
Lipid peroxidation (4-HNE) [78]	Histone adduct formation	Histone H3/H4	Chromatin instability
Mitochondrial dysfunction [52,55]	miRNA dysregulation	miR-200 family, miR-21	EMT, fibrosis, invasiveness
OS [87]	ncRNA processing impairment	Drosha, Dicer	Altered post-transcriptional control

Abbreviations: OS, oxidative stress; ROS, reactive oxygen species; 8-oxo-dG, 8-oxo-2′-deoxyguanosine; DNMT1, DNA methyltransferase 1; HOXA10, Homeobox A10; PGR, progesterone receptor; HDAC1/2, histone deacetylases 1 and 2; NF-κB, nuclear factor kappa B; 4-HNE, 4-hydroxynonenal; miRNA, microRNA; miR-200, microRNA-200 family; miR-21, microRNA-21; ncRNA, non-coding RNA; EMT, epithelial–mesenchymal transition; Drosha, ribonuclease III enzyme Drosha; Dicer, ribonuclease III enzyme Dicer.

**Table 3:** Microbiome alterations and associated molecular mechanisms contributing to inflammation, estrogen metabolism, and lesion progression in EM.

Biological Domain	Key Microbial/Molecular Findings	Mechanistic Impact on EM
Reproductive-tract microbiome [115,116,117]	↓ *Lactobacillus crispatus*/*jensenii*, ↑ *Gardnerella*, *Streptococcus*, *Escherichia*/*Shigella*	Increased epithelial permeability, TLR activation, chronic pelvic inflammation
Gut dysbiosis [105,113,118]	↑ Proteobacteria, Enterobacteriaceae; ↓ Bifidobacterium, Lactobacillus	Systemic inflammation, immune dysregulation, ↑ β-glucuronidase
Estrobolome activity [119,120]	β-glucuronidase-producing bacteria → estrogen deconjugation	Supports estrogen-dependent lesion proliferation
Immune dysregulation (TLR4 axis) [110]	LPS translocation → macrophage activation → IL-6, IL-8, TNF-α	Chronic inflammation, neuroangiogenesis, enhanced lesion invasiveness
SCFA–epigenetic interactions [19]	↓ Roseburia/Faecalibacterium → ↓ butyrate (HDAC inhibitor)	Aberrant DNA methylation, loss of immune tolerance, pro-inflammatory transcription
Microbiome–ROS interplay [121]	Dysbiotic taxa induce ROS; ROS modify DNMT/HDAC activity	Reinforced inflammation, pro-survival lesion phenotype
Infection hypothesis [110]	LPS/flagellin/PGN activate NF-κB → COX-2 → PGE_2_ → aromatase	Initiation or amplification of inflammatory–estrogenic environment
Gut–EM axis [106,122]	Integration of microbial, immune, endocrine alterations	Unified microbiome–immune–estrogen–epigenetic model

Abbreviations: EM, endometriosis; TLR, Toll-like receptor; LPS, lipopolysaccharide; IL, interleukin; TNF-α, tumor necrosis factor-alpha; SCFA, short-chain fatty acid; ROS, reactive oxygen species; DNMT, DNA methyltransferase; HDAC, histone deacetylase; NF-κB, nuclear factor kappa B; COX-2, cyclooxygenase-2; PGE_2_, prostaglandin E_2_; PGN, peptidoglycan.

**Table 4:** Integrated systems-biology model illustrating the bidirectional interactions among epigenetic dysregulation, OS, and microbiome alterations in EM.

Pathophysiologic Domain	Key Mechanisms	Bidirectional Interactions	Resulting Biological Outcomes
Epigenetic Dysregulation [50,54]	DNA hypermethylation (HOXA10, PGR-B, ESR1) Aberrant histone modifications (HDAC ↑, EZH2 ↑) miRNA deregulation (miR-21 ↑, miR-135a/b ↓, miR-200 family ↓, miR-451 ↑)	OS alters DNMT/HDAC activity → epigenetic remodeling Microbial metabolites (SCFAs ↓) reduce HDAC inhibition → pro-inflammatory transcription LPS and cytokines modify miRNA expression	Progesterone resistance Altered estrogen signaling Enhanced proliferation, EMT, fibrosis Impaired endometrial receptivity
OS (ROS Imbalance) [13,130]	Iron overload & Fenton chemistry Activated macrophages (TNF-α, IL-1β, IL-6) Mitochondrial dysfunction ROS-induced DNA, lipid, protein damage	ROS modify methylation patterns & histone structure ROS-driven inflammation alters gut & vaginal microbiota Dysbiosis activates TLR4 → more ROS	Chronic inflammation Neuroangiogenesis & pain sensitization Lesion survival, invasion, angiogenesis Reduced fertility
Microbiome Dysbiosis [102,130]	↓ Lactobacillus; ↑ Gardnerella, Streptococcus, Escherichia/Shigella Gut dysbiosis: ↑ Proteobacteria, Enterobacteriaceae; ↓ SCFA-producing taxa β-glucuronidase-mediated estrogen recycling (estrobolome)	LPS → TLR4 activation → cytokines → ROS ↑ Reduced SCFAs weaken HDAC inhibition → epigenetic dysregulation Estrogen recycling reinforces estrogen-dependent lesion growth	Pelvic inflammation Enhanced estrogen load Increased lesion implantation & persistence Systemic immune activation
Integrated Axis (Systems Interaction) [131]	Microbiome → immune activation → ROS → epigenetic remodeling → altered hormone signaling	Positive feedback loops maintain chronicity	Sustained lesion growth, symptom severity, recurrence, treatment resistance

## Data Availability

No new data were created or analyzed in this study.

## Abbreviations

The following abbreviations are used in this manuscript:

EM	Endometriosis
ROS	Reactive oxygen species
OS	Oxidative stress
HOXA10	Homeobox A10
PGR-B	Progesterone receptor B
ESR1 (Era)	Estrogen receptor alpha
HDAC1/2	Histone deacetylases 1 and 2
H3K27me3	Trimethylation of histone H3 lysine 27
H3K9ac	Acetylation of histone H3 lysine 9
H4K16ac	Acetylation of histone H4 lysine 16
EZH2	Enhancer of zeste homolog 2
EMT	Epithelial–mesenchymal transition
DNMR(s)	Differentially methylated regions
miRNA	MicroRNA
SCFA	Short-chain fatty acid
LPS	Lipopolysaccharide
TLR	Toll-like receptor
COX-2	Cyclooxygenase-2
PGE2	Prostaglandin E2
VEGF	Vascular Endothelial Growth Factor
MMP	Matrix Metalloproteinase
TIMP	Tissue Inhibitor of Metalloproteinases
BPA	Bisphenol A
TCDD	2,3,7,8-Tetrachlorodibenzo-p-dioxin
EDCs	Endocrine-disrupting chemicals
